# Trends in Costs of Birth Hospitalization and Readmissions for Late Preterm Infants

**DOI:** 10.3390/children8020127

**Published:** 2021-02-10

**Authors:** Rebecca R. Speer, Eric W. Schaefer, Mahoussi Aholoukpe, Douglas L. Leslie, Chintan K. Gandhi

**Affiliations:** 1 Pennsylvania State University College of Medicine, 500 University Drive, Hershey, PA 17033, USA; rebeccarspeer@gmail.com; 2 Department of Public Health Sciences, Pennsylvania State University College of Medicine, 500 University Drive, Hershey, PA 17033, USA; eSchaefer@psh.psu.edu (E.W.S.); DLeslie@pennstatehealth.psu.edu (D.L.L.); 3 Center for Host defense, Inflammation, and Lung Disease (CHILD) Research, Department of Pediatrics, Pennsylvania State University College of Medicine, 500 University Drive, P.O. Box 850, H085, Hershey, PA 17033, USA; Aholoukpe.Mahoussi@gmail.com

**Keywords:** preterm birth, resource utilization, birth hospitalization cost, readmission cost, late preterm newborns, term newborns, length of stay

## Abstract

*Background:* The objective is to study previously unexplored trends of birth hospitalization and readmission costs for late preterm infants (LPIs) in the United States between 2005 and 2016. *Methods:* We conducted a retrospective analysis of claims data to study healthcare costs of birth hospitalization and readmissions for LPIs compared to term infants (TIs) using a large private insurance database. We used a generalized linear regression model to study birth hospitalization and readmission costs. *Results:* A total of 2,123,143 infants were examined (93.2% TIs; 6.8% LPIs). The proportion of LPIs requiring readmission was 4.2% compared to 2.1% of TIs, (*p* < 0.001). The readmission rate for TIs decreased during the study period. LPIs had a higher mean cost of birth hospitalization (25,700 vs. 3300 USD; *p* < 0.001) and readmissions (25,800 vs. 14,300 USD; *p* < 0.001). For LPIs, birth hospitalization costs increased from 2007 to 2013, and decreased since 2014. Conversely, birth hospitalization costs of TIs steadily increased since 2005. The West region showed higher birth hospitalization costs for LPIs. *Conclusions:* LPIs continue to have a higher cost of birth hospitalization and readmission compared to TIs, but these costs have decreased since 2014. Standardization of birth hospitalization care for LPIs may reduce costs and improve quality of care and outcomes.

## 1. Introduction

In 2005, the National Institute of Child Health and Human Development recommended replacing the phrase “near term” with “late preterm ” to distinguish infants born between 34 weeks and 0/7 days and 36 weeks and 6/7 days from term infants (TIs) (≥37 weeks), acknowledging the vulnerability of the late preterm infants (LPIs) and helping redirect research efforts [1]. The earlier phrase “near term” was ambiguous and underestimated inherited risks such as respiratory distress, temperature instability, hypoglycemia, poor feeding, and hyperbilirubinemia at birth for LPIs compared to TIs [1,2,3,4,5,6,7,8]. Late prematurity is also associated with long-term morbidities such as metabolic syndrome, obesity, hypertension, diabetes, poor neurodevelopmental and behavioral outcomes [1,8,9,10,11,12,13,14,15,16,17].

Hospital costs of birth are negatively correlated with gestational age [18]. Moreover, prematurity is associated with increased short- and long-term readmission rates compared to TIs [2,5,7,18,19,20]. The discrepancy in healthcare costs between LPIs and TIs persists throughout the first year of life [4,8,21]. Provisional birth data from the Centers for Disease Control and Prevention (CDC) [22] reported a decrease in total births in the United States (US) but an increase in preterm births in the most recent four years (2014–2018), accounting for roughly 10% of total births in 2018. Despite efforts to ameliorate the costs [19], such as determining gestational age based appropriate length of stay (LOS), the upward trend of late premature births has created a heavy burden in terms of healthcare costs [18,19,21]. Though LPIs constitute only a fraction of all live births, they account for 11.2% of total neonatal costs (1.145 billion USD annually) [23].

The objective of the present study was to examine previously unexplored trends of birth hospitalization and readmission costs for LPIs compared to TIs in the US between 2005 and 2016 using employer-sponsored private health insurance database. We hypothesized that we would observe a decrease in birth hospitalization and readmission costs, and readmission rates, for LPIs during this timeframe.

## 2. Methods

### 2.1. Data Source

The data in this study were extracted from the Truven Health Analytics MarketScan^®^ Commercial Claims and Encounters Database [24], which collects data annually from millions of commercially insured individuals with employer-sponsored private healthcare plans. Data are collected from all census regions of the US, and a unique enrollee identification code is assigned to each individual, allowing for longitudinal tracking of each member. The database contains member’s information such as demographic information, inpatient and outpatient encounters (including diagnosis codes, diagnosis-related group (DRG) codes, LOS, and discharge status), and financial information, among other variables. MarketScan data are de-identified and Health Insurance Portability and Accountability Act compliant.

### 2.2. Study Sample Identification

Infants with birth hospitalizations were identified in calendar years 2005 to 2016 by extracting all inpatient claims that met predetermined inclusion criteria of admission type of “Maternity & Newborn”, age = 0, and relationship of child to the primary beneficiary. We used both International Classification of Diseases (ICD) diagnosis codes and DRG codes to identify that these claims were birth hospitalizations (Supplementary Table S1). Claims with ICD-9 codes of 765.27-28, ICD-10 codes P07.36-39 or DRG codes indicating prematurity (791, 792) on the birth admission claim were classified as LPIs (33–36 weeks). TIs (≥37 weeks) were identified using ICD-9 codes 765.29, 766.21-22, ICD-10 codes P08.21-22, or DRG codes (793–795). For multiple diagnosis codes in birth hospitalization claims, the code reflecting the earliest gestational age was considered. Similarly, for multiple DRG codes, lower numeric values took precedence (e.g., 792 over 793).

We excluded infants without ICD or DRG codes, or a diagnosis code indicating an unspecified week of gestation on birth hospitalization claim. Infants with a diagnosis code indicating <33 weeks gestational age or a DRG code of 790, indicating possible extreme immaturity, were also excluded from the study. Finally, we excluded infants with <30 days of continuous enrollment in MarketScan-covered insurance starting from the date of birth hospitalization discharge to study the trend of readmission costs of LPIs and TIs within 30 days of discharge.

The clinical variables such as mode of delivery and multiple births were determined using ICD codes (Supplementary Table S1). On the initial birth admission, newborns who had a subsequent inpatient admission on the day of discharge were classified as being transferred.

### 2.3. Outcomes

We were primarily interested in studying the trend of healthcare cost of LPIs compared to TIs during birth hospitalization. Costs were measured from the perspective of the healthcare system and included total payments for claims eligible under the medical plan after applying rules such as discounts, including patient out-of-pocket payments. All costs from inpatient claims that occurred during the birth hospitalization were summed to create the total healthcare costs for an infant. All costs were adjusted to 2016 US dollars using the medical component of the consumer price index.

For a small proportion of infants, the total healthcare costs were negative or 0 USD. Negative costs represent reimbursement or other adjustments to a previous error in billing. However, the error cannot be directly determined from the claims in which the reimbursement or adjustment occurred. These negative values are nonsensical for our purposes. To account for these values, we used Winsorization on 1.5% of the data, or 0.75% at each end of the distribution [25,26]. The method set values less than the 0.75th percentile (<0.75%) to the 0.75th percentile, and values greater than the 99.25th percentile (>0.75%) to the 99.25th percentile. For example, the 0.75th percentile for LPIs was 262 USD and the 99.25th percentile was 280,869 USD. Costs less than 262 USD were set to a value of 262 USD and costs greater than 280,869 USD were set to a value of 280,869 USD. Winsorization was applied separately for each group (LPIs and TIs) because the distributions were very different.

Readmissions were defined as inpatient hospitalizations occurring in the first 30 days after discharge from the birth hospitalization. Costs during readmissions were also calculated in the same way as birth hospitalization costs (including Winsorization).

### 2.4. Statistical Analysis

For our primary goal of estimating birth hospitalization costs for LPIs vs. TIs, we used generalized linear regression models with a Gamma distribution and log link [27]. The Gamma distribution was used because costs exhibited skewness, with some infants accruing very large costs. We fit two models. The first included only an indicator for gestational age (LPI vs. TI). The second model included an indicator for gestational age, birth year, and their interaction. The interaction term allowed for different trajectories in costs over time by gestational age groups.

Cost ratios (CRs) and 95% confidence intervals (CIs) were reported for LPIs compared to TIs from these models [28]. While CRs can be directly calculated from the parameters in the model, mean costs are more meaningful. Thus, we used the inverse link function to obtain mean costs on original dollar scale adjusted to 2016 dollars.

Our secondary goals were to examine readmissions rates and costs of readmissions. Readmissions were modeled using logistic regression with odds ratios (ORs) and corresponding 95% CIs reported [28]. Costs of readmissions were modeled using the same generalized linear regression models described above.

Finally, we also examined regional differences in birth hospitalization costs, readmissions, and costs of readmissions. Models were fit separately for each region in the database (Northeast (NE), North central (NC), South (S), and West (W)), with results shown graphically.

## 3. Results

**Figure 1:**
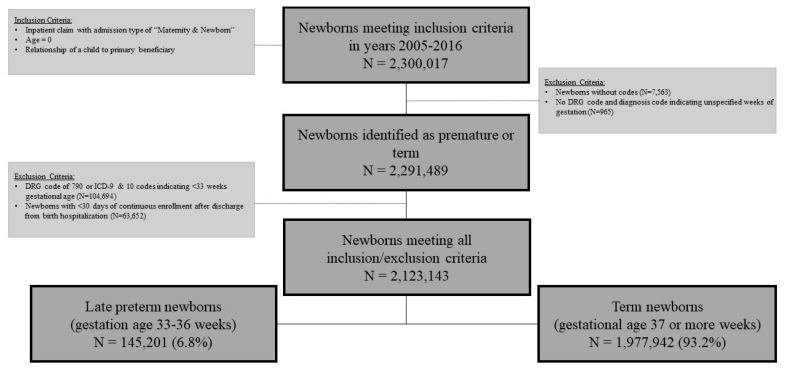
Sample selection: summarization of study sample size.

The total number of infants with birth hospitalizations during the years 2005 to 2016 was 2,300,017. After applying exclusion criteria, the study cohort consisted of 2,291,489 infants. Based on ICD and DRG codes, 2,035,825 infants (88.8%) were classified as term and 255,664 infants (11.2%) were classified as preterm with further sub-classification into <33 weeks (*n* = 104,694 infants, 4.6%) and 33–36 weeks (*n* = 150,970 infants, 6.6%). After applying the continuous 30-day enrollment criterion, the final study cohort consisted of 2,123,143 infants with 145,201 (6.8%) classified as late preterm and 1,977,942 (93.2%) classified as term (Figure 1).

### 3.1. Birth Hospitalization Characteristics

Table 1 shows the infant characteristics of TIs and LPIs in calendar years 2005–2016. Overall, higher proportions of LPIs were born via cesarean deliveries (54.0% vs. 35.0%), were from multiple births (22.5% vs. 1.7%), were transferred from a birth hospital (3.0% vs. 0.4%), were admitted in Neonatal Intensive Care Unit (NICU) (56.7% vs. 7.2%), and had longer LOS (median 4 vs. 2 days) compared to TIs. Although the majority of TIs and LPIs were discharged within 3 days of birth, TIs were twice likely to go home within 3 days compared to LPIs (91.8% vs. 45.6%). Moreover, 23.5%, 28.2% and 2.7% of LPIs were discharged between 4 and 7 days, 8 and 30 days, and ≥31 days after birth, respectively.

### 3.2. Birth Hospitalization Cost

**Figure 2:**
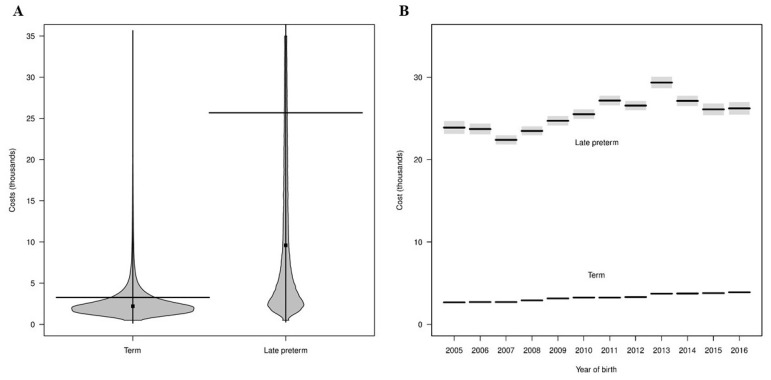
Healthcare costs of birth hospitalization. Panel (**A**) displays a bean plot of distributions of total birth hospitalization costs for term and late preterm infants. The horizontal line indicates the mean value and the dot indicates the median value. Costs greater than 35,000 USD are not displayed. Panel (**B**) displays estimated mean birth hospitalization costs from 2005–2016 from a fitted regression model. Estimated mean costs are indicated by black lines and 95% confidence intervals by gray regions. Gray regions are not observed for term infants because the confidence intervals are very narrow around the mean estimates.

Figure 2A shows the distribution of total healthcare costs of birth hospitalizations displayed as a bean plot. The distributions were highly skewed and indicated that the majority of infants in both cohorts had costs less than 10,000 USD. Additionally, 22% of LPIs had total healthcare costs greater than 35,000 USD compared to only 0.8% of TIs. Mean costs associated with birth hospitalizations were substantially higher for LPIs than TIs (25,700 vs. 3300 USD, Table 2). Based on the fitted regression model that included only an indicator for gestational age, estimated mean costs for LPIs were 7.86-times greater than TIs (95% CI: 7.80 to 7.91), with corresponding mean cost estimates of 25,700 USD (95% CI: 25,500 to 25,900 USD) for LPIs and 3300 USD (95% CI: 3200 to 3400 USD) for TIs.

A regression model was fit to examine the trend of birth hospitalization costs for LPIs and TIs in years 2005–2016. Estimated CRs and mean costs from the model are shown in Table 3 and Figure 2B, respectively. The mean cost for LPIs was lowest (22,400 USD) in 2007 and highest (29,400 USD) in 2013. In general, the mean costs associated with late preterm births decreased from 2005 to 2007 (23,900 to 22,400 USD), with an overall increase until 2013 (highest), followed by mostly decreased mean costs from 2014 to 2016 (see Figure 2B). In contrast, for TIs, mean costs increased steadily from 2005 to 2016 (2670 to 3890 USD). CRs (LPIs vs. TIs) trended downward from 2005 to 2016 (from 8.94 to 6.73) due to the relative increase in birth hospitalization costs for TIs compared to LPIs (Table 3).

**Figure 3:**
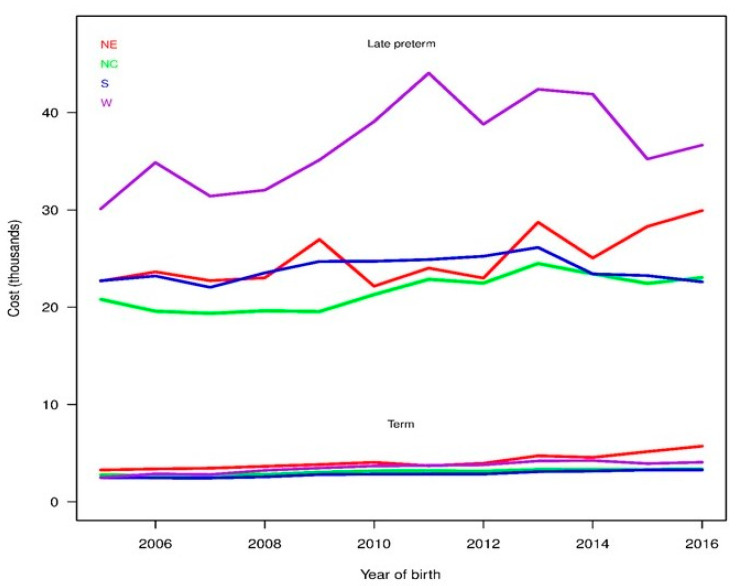
Birth hospitalization costs based on region. Estimated mean birth hospitalization costs for late preterm and term infants from regression models fitted separately for each region. The United States was divided in four representative regions (Northeast (NE), Northcentral (NC), South (S), and West (W).

Figure 3 shows mean birth hospitalization costs from fitted regression models for LPIs and TIs based on four US regions. In general, the trend remained the same for each region from 2005 to 2016 (primarily increasing costs over time, with some decreases since 2013). Of interest, birth hospitalization cost of TIs were similar in all four regions, whereas, the West region consistently showed considerably higher cost for LPIs compared to other three regions (NE, NC, S). We observed lower CRs with narrow CIs for LPIs in the NE region compared to the West region (higher CRs with wide CIs, data not shown).

### 3.3. Readmissions Rates and Costs

**Figure 4:**
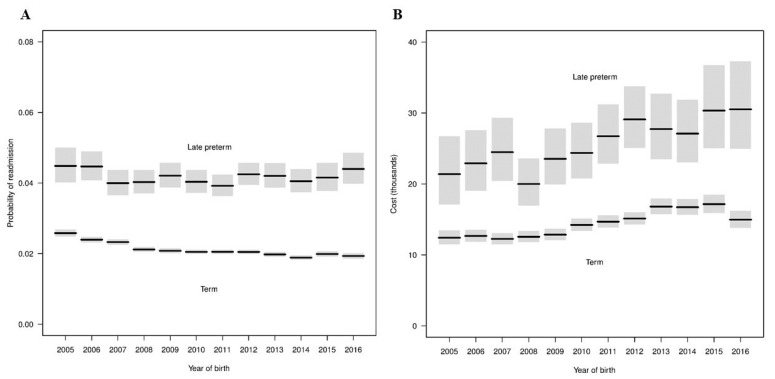
Readmission rates and costs for late preterm and term infants from 2005–2016. Panel (**A**) displays estimated probabilities indicated by black lines and 95% confidence intervals by gray regions from fitted regression model for readmissions. Panel (**B**) displays estimated mean costs indicated by black lines and 95% confidence intervals by gray regions from fitted regression model for readmission costs.

The percentage of LPIs readmitted within 30 days of discharge from birth hospitalization was twice that of TIs (4.2% vs. 2.1%). Based on the fitted regression model that included only gestational age, LPIs had 2.02-times (95% CI: 1.97–2.08, *p* < 0.001) higher odds of readmission compared to TIs. We also examined readmissions for LPIs and TIs in calendar years 2005–2016 using a logistic regression model. Estimated probabilities from the model are shown in Figure 4A. The interaction term in the model was significant (*p* = 0.002), indicating that the proportion of infants with a readmission differed across calendar year by cohorts. For TIs, readmission rates decreased significantly (*p* < 0.001) over the years. However, we did not observe a significant difference in trend of readmission rate across time for LPIs (*p* = 0.50). We observed a general trend of increasing estimated ORs of readmissions for LPIs compared to TIs from 1.77 in 2005 to 2.33 in 2016 (Table 4) and it is due to relative decrease in readmissions for TIs compared to LPIs.

The analysis of readmission costs was restricted to the subgroup of 47,543 infants with a readmission (6030 LPIs and 41,513 TIs). The LPIs had higher median (9300 vs. 6800 USD) and mean (25,800 vs. 14,300 USD) readmission costs than TIs. Based on the fitted regression model that included only gestational age, estimated mean readmission costs for LPIs were 1.80-times greater than TIs (95% CI: 1.70 to 1.89), with corresponding mean cost estimates of 25,800 (95% CI: 24,500 to 27,100 USD) for LPIs and 14,300 (95% CI: 14,100 to 14,600 USD) for TIs.

**Figure 5:**
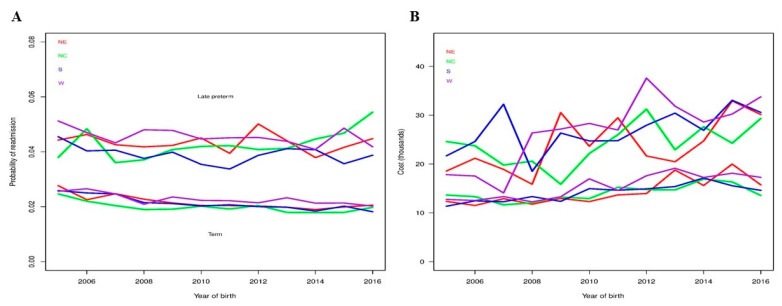
Readmission rates and costs for late preterm and term infants from 2005–2016. Panel (**A**) displays estimated probabilities of readmission for late preterm infants (LPIs) compared to term infants (TIs) by region based on fitted regression models. Panel (**B**) displays estimated mean costs by region from fitted regression models.

A regression model was fit to examine the readmission costs for LPIs and TIs in calendar years 2005–2016. Estimated CRs for readmission costs from the model are shown in Table 3. The interaction between cohort and birth year was not significant (*p* = 0.75), indicating that mean cost estimates over time did not differ significantly by cohort. As shown in Figure 4B, mean costs generally increased for each group from 2005 to 2016. The mean costs for LPIs increased from 21,400 USD in 2005 to 30,500 USD in 2016 with a wide confidence interval. Conversely, we observed a steady trend of increasing mean readmission costs of TIs from 12,400 USD in 2005 to 17,200 USD in 2015 and then decreased slightly to 15,000 USD in 2016. Generally, the mean costs of readmission increased by approximately 3% in each group. Of note, readmission rates and costs for LPIs and TIs were broadly similar among four regions during the study period (Figure 5).

## 4. Discussion

Although the phrase “late preterm” was introduced in 2005, we continued to observe a trend of increased birth hospitalization costs for LPIs compared to TIs until 2013. Of note, we observed a recent decline in birth hospitalization costs of LPIs from the year 2014. In contrast, the birth hospitalization cost of TIs has steadily been increasing since 2005. Our study also demonstrates that LPIs were twice as likely to be readmitted compared to TIs. We observed a stable readmission rate for LPIs but a steady decline in readmission rate for TIs between 2005 and 2016. The readmission cost has steadily increased by approximately 3% for both LPIs and TIs since 2005. The West region showed a higher birth hospitalization costs for LPIs compared to other regions, whereas, no major geographical variation was noted in birth hospitalization costs for TIs and readmission costs for LPIs and TIs.

Our study confirmed previous findings of higher birth hospitalization costs for LPIs compared to TIs regardless of study design, costing methodology, location and year of study [21,29,30,31]. Studies conducted in the US showed 7–12-fold (average 7.8-fold in the present study) higher cost of initial hospitalization [21,32,33] compared to similar studies from other developed countries showing ~2–4-fold increase in initial hospitalization cost for LPIs [29,34,35]. A previous study examining initial hospitalization costs of a 1998–2000 California birth cohort showed six-fold higher costs of LPIs [36]. However, the study used 37 week infants as a control instead of term births; hence, the authors may have underestimated late preterm birth hospitalization cost. A study of a 2004 birth cohort using private insurance showed almost 12-times higher cost of birth hospitalization for LPIs compared to TIs [21]. In line with this finding, we observed 8.94-times higher cost of late preterm birth hospitalization in 2005 (highest) compared to TIs and decreasing differential cost thereafter, reaching 6.73-times in 2016 (lowest). This trend of decreasing differential cost for late preterm births is due to the combined effect of both increasing birth hospitalization cost of TIs along with decreasing cost for LPIs, at least since 2014. As shown in Figure 2B, the birth hospitalization cost for LPIs peaked in 2013. We studied the trend of maternal and neonatal variables such as proportion of multiple births, cesarean delivery, NICU stay, inter-hospital transfer and LOS that may have an impact on trend of birth hospitalization cost. We did not observe any particular trend in any of the studied variables (data not shown) except for LOS (decreased after 2013), which may explain decreasing cost of LPIs since 2013. Of note, due to limitations of administrative database, we were not able to adjust for other variables that may have an impact on birth hospitalization cost, e.g., maternal conditions and/or complications, social factors such as administrative discharge hold for non-medical reasons. Future prospective studies are needed to investigate the impact of these variables on neonatal costs. The recent trend of decreasing birth hospitalization cost for LPIs may be due to either heightened awareness among providers for this vulnerable cohort or natural variation in the trends. However, it is more likely due to the former because similar trends of decreasing NICU utilization have been observed in recent population studies from the US [37,38,39]. The other possibility is introduction of the Children’s Health Insurance Program and the Affordable Care Act (ACA) in 2009 and 2010, respectively [40]. Lower inpatient Medicare hospital payment rates as a part of ACA led to a spillover reduction in private insurance rates [41]. Though the ACA has slowed the growth of healthcare spending in the last decade, inpatient costs increased by ~4% yearly without any change in LOS [42]. We observed a similar trend of increasing birth hospitalization cost of TIs by 3% during the studied period without any change in LOS but decreasing birth hospitalization cost for LPIs since 2013. Nonetheless, there is still a potential to reduce birth hospitalization cost for LPIs in the US considering other developed countries report lower differential birth hospitalization cost (~2–4 fold) for LPIs [29,34,35].

Our study also showed a 2x higher readmission rate for LPIs compared to TIs. For the current study, we focused on readmission rate and inpatient cost within 30 days of discharge from birth hospitalization for two reasons: (i) inpatient costs are the largest contributor (~92%) of the economic implications of preterm birth costs [43] and (ii) the risk of readmission is the highest within 30 days of discharge [7,44,45]. In our study, the OR of readmission increased from 1.7 in 2005 to 2.3 in 2016 for LPIs compared to TIs. As shown in Figure 4A, this steady increase in OR is due to the same rate of readmission for LPIs since 2005 but decreasing readmissions for TIs. Several past studies have shown 1.5- to 3-times higher readmission for LPIs compared to TIs [7,20,21,44]; however, the duration of follow up ranges from 7 days to early childhood [19,43,44,46]. Our results showed that medical advances and better understanding of need of LPIs in the last decade did not result in decrease readmissions for LPIs. Surprisingly, the readmission rate of TIs decreased consistently during the study period. This has never been reported before. In contrast, a recent study from Canada showed increasing readmission rates for TIs from 2003 to 2012 [47]. This unique finding of the current study needs further exploration to evaluate causes and/or factors responsible for decreasing readmission in TIs and may apply the same principles to decrease readmission rates in LPIs in the future. Of note, neonatal jaundice remained the number one cause of readmissions for LPIs and TIs (data not shown). In the current study, LPIs had on average 1.8-times higher cost of readmissions than TIs. These data indicate that not only are LPIs at high risk for readmission, they are also susceptible to develop more severe illness and require more medical services. In addition, we observed a steady trend of increasing readmission cost by 3% for both LPIs and TIs. As mentioned above, there is a trend of increasing inpatient healthcare cost by ~4% in last decade [42]; hence, our findings are not surprising. In addition, we observed a wide variation in birth hospitalization costs, but not in readmission costs, of LPIs based on the region, particularly the West region showed consistently higher birth hospitalization costs for LPIs. Factors such as differences in patient health status, treatment preferences, physician practice patterns, access to and availability of services, and wages/cost of living may help explain these types of geographic variation [48,49]. However, it is important to note that the birth hospitalization costs of TIs and readmission costs of LPIs and TIs remained similar across regions during study period, Figure 3 and Figure 4. Together, these data indicate that standardization of birth hospitalization care of LPIs, particularly admission and discharge criteria, across regions may reduce costs for LPIs and in turn improve quality of care and outcomes [50].

Our study has several limitations. First, our data include only commercially insured infants with employer-sponsored private healthcare plans; thus findings cannot be generalized to infants insured through Medicaid or State Children’s Health Insurance Programs. Of note, private insurance inpatient hospital payment rates follow the government insurance inpatient payment rates [41]. Therefore, we expect to observe a similar trend of initial and readmission hospitalization costs for LPIs for government sponsored insurance plan as well, but need additional research to confirm this speculation. Second, claim-based data do not provide important clinical information such as ethnicity, breast feeding status, socioeconomic status, and individual hospital practices that may impact birth hospitalization costs. Third, ICD-9 codes do not differentiate between infants born at 33- and 34-weeks’ gestation. Thus, some of our data fell outside the current definition of late-preterm (34–36 weeks gestation). Additionally, the accuracy of ICD-9 and -10 codes depends on the diagnosis, with sensitivity ranges from 45% to 90% with high specificity; thus some of the eligible infants could have been missed [51]. Healthy infants born at 35- to 36-weeks’ gestation are less likely to be coded as LPIs and are more likely to have shorter LOS than sick LPIs of the same gestation [33], causing overestimation of LPIs costs. However, it is important to note that validity of ICD-9 codes has already been established for gestational age and birthweight [52] and the ICD-10 codes are considered to be more reliable [53]. More importantly, our study cohort consisted of ~7% LPIs and ~88.8% Tis—numbers similar to the CDC’s prematurity data of the last decade. Therefore, we believe that we have reliably captured the majority of eligible LPIs, and missing or misclassified infants would not have changed our study findings considering the large sample size of ~2,300,000 infants included for analysis.

## 5. Conclusions

In summary, we continue to observe higher birth hospitalization costs, readmission rate and costs for LPIs compared to TIs. Moreover, there is a recent trend of decreasing birth hospitalization costs for LPIs, but the cost of readmission is still increasing. There is a consistent trend of decreasing readmission rate for TIs but birth hospitalization and readmission costs have increased over last decade. Given recent evidence of an increasing incidence of preterm birth, information on trends in the costs allow analysts and policymakers to plan clinical and budgetary services for the future. In addition, our data should be of interest to other researchers that are planning to evaluate the cost-effectiveness of new prevention and treatment strategies for LPIs.

## Figures and Tables

**Table 1:** Infant characteristics stratified by gestational age.

	Term (*N* = 1,977,942)	Late Preterm (*N* = 145,201)
**Birth year**		
2005	92,483 (4.7%)	6690 (4.6%)
2006	139,575 (7.1%)	9736 (6.7%)
2007	159,278 (8.1%)	11,434 (7.9%)
2008	180,230 (9.1%)	13,482 (9.3%)
2009	178,219 (9.0%)	12,662 (8.7%)
2010	189,559 (9.6%)	14,402 (9.9%)
2011	211,864 (10.7%)	15,734 (10.8%)
2012	220,843 (11.2%)	15,938 (11.0%)
2013	171,590 (8.7%)	12,921 (8.9%)
2014	180,984 (9.2%)	13,994 (9.6%)
2015	132,868 (6.7%)	9774 (6.7%)
2016	120,449 (6.1%)	8434 (5.8%)
**Delivery**		
Vaginal	1,284,880 (65.0%)	66,794 (46.0%)
Cesarean	693,062 (35.0%)	78,407 (54.0%)
**Multiple birth**		
No	1,945,008 (98.3%)	112,575 (77.5%)
Yes	32,934 (1.7%)	32,626 (22.5%)
**Transfer**		
No	1,970,651 (99.6%)	140,852 (97.0%)
Yes	7291 (0.4%)	4349 (3.0%)
**NICU stay**		
No	1,834,790 (92.8%)	62,832 (43.3%)
Yes	143,152 (7.2%)	82,369 (56.7%)
**LOS, days**		
Median	2	4
Interquartile range	2, 3	2, 9
**LOS, days (group)**		
≤3 days	1,816,309 (91.8%)	66,273 (45.6%)
4–7 days	144,338 (7.3%)	34,160 (23.5%)
8–30 days	15,993 (0.8%)	40,910 (28.2%)
≥31 days	1302 (0.1%)	3858 (2.7%)

Abbreviations: LOS—length of stay, NICU—Neonatal Intensive Care Unit.

**Table 2:** Summary of birth hospitalization costs.

Summary of Birth Hospitalization Costs (USD)			
	Term (*N* = 1,977,942)	Late Preterm (*N* = 145,201)	Total (*N* = 2,123,143)
Cost of birth hospitalization			
Mean (SD)	3300 (4400)	25,700 (41,400)	4800 (13,000)
Median	2200	9600	2300
Interquartile range	(1600–3200)	(3500–30,200)	(1600–3400)

*N* = number, SD = standard deviation. Costs were rounded to the nearest 100 USD.

**Table 3:** Cost Ratios (late preterm vs. term infants) from fitted regression model for birth hospitalization and readmission costs from 2005 to 2016.

Parameter	Birth Hospitalization Costs CR (95% CI)	Readmission Costs CR (95% CI)	*p* Value
Late preterm vs. term by year			
2005	8.94 (8.64–9.25)	1.72 (1.36–2.18)	<0.001
2006	8.76 (8.52–9.01)	1.80 (1.48–2.20)	<0.001
2007	8.28 (8.07–8.49)	2.00 (1.65–2.42)	<0.001
2008	8.07 (7.88–8.27)	1.59 (1.33–1.90)	<0.001
2009	7.85 (7.66–8.05)	1.83 (1.53–2.19)	<0.001
2010	7.84 (7.66–8.02)	1.71 (1.44–2.03)	<0.001
2011	8.37 (8.18–8.55)	1.82 (1.54–2.15)	<0.001
2012	8.02 (7.85–8.20)	1.92 (1.64–2.26)	<0.001
2013	7.88 (7.69–8.07)	1.65 (1.38–1.97)	<0.001
2014	7.24 (7.07–7.41)	1.62 (1.36–1.93)	<0.001
2015	6.89 (6.70–7.09)	1.77 (1.44–2.17)	<0.001
2016	6.73 (6.53–6.94)	2.04 (1.64–2.53)	<0.001

Abbreviation: CI—Confidence Interval.

**Table 4:** Odds ratios from fitted regression model for readmissions from 2005 to 2016.

Odds Ratios (OR) from Fitted Regression Model for Readmissions from 2005 to 2016	
Parameter	OR (95% CI)
Late preterm vs. term by year	
2005	1.77 (1.57–2.00)
2006	1.90 (1.72–2.11)
2007	1.75 (1.58–1.93)
2008	1.94 (1.77–2.13)
2009	2.07 (1.88–2.27)
2010	2.01 (1.84–2.20)
2011	1.95 (1.79–2.12)
2012	2.12 (1.95–2.30)
2013	2.17 (1.98–2.38)
2014	2.19 (2.00–2.40)
2015	2.13 (1.92–2.37)
2016	2.33 (2.09–2.61)

## Data Availability

Restrictions apply to the availability of these data. Data was obtained from Truven Health MarketScan Database and are available https://www.ibm.com/products/marketscan-treatment-pathways with the permission.

## Supplementary Materials

The following are available online at https://www.mdpi.com/2227-9067/8/2/127/s1, Table S1: International Classification of Diseases (ICD) and Current Procedural Terminology (CPT) codes for clinical variables.
