# Supplementary material for: Trends in Costs of Birth Hospitalization and Readmissions for Late Preterm Infants
Source: Children (Basel). 2021 Feb 10;8(2):127. doi: 10.3390/children8020127 (PMC7916486; doi:10.3390/children8020127)
Supplement: Supplementary file 1 [file children-08-00127-s001.pdf]

Supplementary Table 1: International Classification of Diseases (ICD) and Current Procedural Terminology (CPT) codes for clinical variables

Variables Based on Claims for Birth Hospitalization		
	**ICD-9 Diagnosis Codes**	**ICD-10 Diagnosis Codes**
	**V30.xx-V39.xx**	**Z38.xx**
**Delivery Method**		
Vaginal	V30.xx-V39xx with 5th digit: 0	Z38.61 and Z38.xx with 5th digit: 0, 3, 5, 8
Cesarean	V30.xx-V39xx with 5th digit: 1	Z38.xx with 5th digit: 1, 2, 4, 6, 9
**Number of Deliveries**		
Singleton	All other codes indicate singleton	Z38.00, Z38.01, Z38.1, and Z38.2
Multiple	V31.xx to V37.xx	All other codes indicate multiple
**NICU Stay codes**	Inpatient claim code type of 61 (Intensive care unit)<br><br>Revenue center codes: 0172-0175, 0203<br>CPT codes: 99468, 99471, 99477, 99469, 99472, 99479, 99480	
